# Optimizing Ammonia Separation from Thermophilic Digestate: The Combined Effect of pH and Thermal Gradients in Direct Contact Membrane Distillation

**DOI:** 10.3390/membranes15120348

**Published:** 2025-11-22

**Authors:** Fanny Rivera, Luis Villarreal, Pedro Prádanos, Raúl Muñoz, Laura Palacio, Antonio Hernández

**Affiliations:** 1 Institute of Sustainable Processes, University of Valladolid, 47011 Valladolid, Spain; fannymaritza.rivera@uva.es (F.R.); luis.villareal@uva.es (L.V.); ppradanos@uva.es (P.P.); raul.munoz.torre@uva.es (R.M.); laura.palacio@uva.es (L.P.); 2 Department of Applied Physics, Science Faculty, University of Valladolid, 47011 Valladolid, Spain; 3 Department of Chemical Engineering and Environmental Technology, University of Valladolid, 47011 Valladolid, Spain

**Keywords:** circular economy, distillation, flat-sheet membrane module, TAN recovery, thermophilic anaerobic sludge

## Abstract

Ammonia recovery from synthetic thermophilic anaerobic digestate was achieved through Direct Contact Membrane Distillation (DCMD) using hydrophobic flat-sheet membranes under different operating conditions. The influence of temperature gradients (0 °C, 20 °C, 35 °C, and 45 °C) and pH levels of the thermophilic anaerobic sludge (7.8, 8.2, 9, and 12) was investigated. The process utilized a DCMD setup with hydrophobic PTFE membranes of 0.22 µm nominal pore radius, and receiving solutions consisting of deionized water and 1 M H_2_SO_4_. The best results were obtained with isothermal distillation and high pH levels in the feed. Isothermal distillation at 65 °C (a temperature gradient of 0 °C), with 1 M H_2_SO_4_ as the receiving solution, and at pH levels 8.2 and 12, yielded NH_3_ recoveries of 36.4 ± 1.6% and 100.0 ± 0.1%, respectively. Under the same conditions, the molar fluxes were 0.63 ± 0.01 mol TAN m^−2^ h^−1^ and 1.84 ± 0.01 mol TAN m^−2^ h^−1^, respectively. It is worth noting that some very low depositions on the membrane were detected, leading to changes in the surface morphology, as confirmed by atomic force microscopy.

## 1. Introduction

Ammonia (NH_3_) is a colorless gas with a pungent odor, highly soluble in water, and vital in biological and industrial processes. It plays a central role in producing fertilizers, explosives, and cleaning agents, and is naturally released through the decomposition of organic matter and nitrogen metabolism. NH_3_ significantly influences atmospheric chemistry and air–surface exchanges, with profound implications for ecosystems and human health [1].

While NH_3_ can boost crop yields in nitrogen-deficient areas, excessive emissions in regions with high nitrogen availability can damage ecosystems. Poor management of animal manure and crop residues intensifies nitrogen release, which, when deposited via rainfall, can lead to the eutrophication and acidification of water bodies. NH_3_ reacts in the atmosphere to form fine particulate matter (PM2.5), such as ammonium sulfate and ammonium nitrate, contributing to air pollution and health risks, including respiratory problems and premature deaths [1,2].

Agriculture is responsible for nearly 90% of global NH_3_ emissions, mainly from ammonia-based fertilizers and livestock manure [3]. Annually, around 54 Mt of nitrogen-ammonia are emitted, with human activities accounting for roughly 60% [4]. Additionally, NH_3_ emissions serve as precursors for nitrous oxide (N_2_O), a potent greenhouse gas associated with health risks like respiratory issues and cancer [5].

Given its widespread environmental impacts, effective NH_3_ emission control requires global cooperation and strong policy frameworks. Regulations have been established in several regions: the U.S., EU, and China have set discharge standards for ammonia nitrogen in surface water, ranging from 1.5 to 17 mg L^−1^ [6]. The EU’s Directive 2016/2284 (Table B, page 20) mandates a reduction in NH_3_ emissions by up to 3% from 2020–2029 and up to 16% from 2030 onwards [7].

Wastewater contains various pollutants, including nutrients, pathogens, suspended solids, and organic and inorganic matter. Its proper treatment is crucial for environmental protection, public health, and water resource sustainability. Wastewater and sludge management involves collecting, treating, and safely disposing of or reusing contaminated water and byproducts. Effective treatment removes pollutants, safeguarding water bodies from contamination and preventing ecological degradation [8].

Sludge, a byproduct of wastewater treatment, must be managed carefully due to its high organic content and potential hazards. Advanced treatment technologies like anaerobic digestion and thermal hydrolysis stabilize sludge, reduce its volume, and generate valuable byproducts such as biogas and biosolids that can be reused as fertilizers, improving soil fertility and reducing reliance on synthetic fertilizers [9].

Emerging technologies, including membrane bioreactors and advanced oxidation processes, further enhance wastewater and sludge treatment, promoting resource recovery and supporting energy sustainability [10]. Sustainable solutions like anaerobic digestion and bioremediation can convert agricultural waste into usable products, minimizing waste and improving resource efficiency [11]. Improving wastewater treatment and sludge reuse will help address environmental contamination and support sustainable agriculture by closing nutrient loops and recovering valuable resources.

Currently, various technologies are used to reduce nitrogen in livestock wastewater, including electrochemical cells, stripping, nitrification–denitrification, ion exchange, zeolite adsorption, partial nitritation-anammox, and gas-permeable membranes [12,13]. Gas-permeable membranes extract NH_3_ by allowing it to pass through a hydrophobic membrane into an acidic solution, typically sulfuric, phosphoric, or nitric acid, producing fertilizers like ammonium sulfate, ammonium phosphate, and ammonium nitrate [14,15]. This method operates without high pressures, extensive pretreatment, or high energy demands, offering a cost-effective and sustainable approach aligned with circular bioeconomy principles.

From an operational standpoint, the most promising membrane methods for ammonia recovery are those that treat a gas stream, those based on electrokinetic phenomena, and those employing Direct Contact Membrane Distillation (DCMD). All these methods have achieved high efficiency in ammonia recovery. In the case of processes that treat a gas stream, those utilizing membranes highly selective to NH3 (such as perfluorinated sulfonic acid (PFSA) membranes) offer very good performance and result in a high-purity byproduct [16]. However, when ammonia is dissolved in the liquid phase, this approach requires a stripping process, and the membrane must be periodically regenerated. Regarding methods based on electrokinetic phenomena, examples include electrodeionization, which is a hybrid method combining ion exchange with electrodialysis. This also necessitates coupling with a stripping process. The examples studied demonstrate the feasibility of the process, but the proposed system, particularly on a large scale, is often too complex [17]. DCMD systems are simpler from a process technology perspective, although they require an energy input to accelerate the mass transfer process. However, the main energy input in this type of process can be easily obtained with solar thermal panels [18]. In a previous study [19], the energy consumption for a full-scale plant of this type, utilizing poultry manure as the nitrogen source, was calculated. The consumption was 0.48 kWh kg^−1^ of recovered N (without considering the use of green energy). This is significantly lower than a traditional process, such as ammonia stripping, which can require up to 8.65 kWh kg^−1^ of recovered N.

Membrane Distillation (MD), effective for separating volatile compounds, has gained attention for treating NH_3_-rich wastewater. This thermally driven process uses a porous, hydrophobic membrane to maintain a liquid–vapor interface, preventing feed solutions from penetrating the permeate side. The vapor pressure difference across the membrane, often generated using low-grade heat sources like solar energy, drives the process efficiently [20]. Unlike pressure-driven filtration, MD operates near atmospheric pressure, reducing fouling risks [21,22]. The absence of osmotic pressure and low sensitivity to concentration polarization enable significant volume reduction and high-water recovery [23,24]. However, when recovering NH_3_ from dilute streams, the permeate is diluted by water vapor, and the maximum NH_3_ concentration is limited by the NH_3_-to-water flux ratio [25]. Critical factors for NH_3_ recovery in MD include temperatures, flow rates, NH_3_ concentration, feed pH, and the nature of the distillate (H_2_O or acid) [26]. Raising the feed pH from 10.0 to 11.8 can increase NH_3_ recovery up to 93% [27].

In this study, the Direct Contact Membrane Distillation (DCMD) process was evaluated for NH_3_ recovery using thermophilic anaerobic sludge as model wastewater. High total ammoniacal nitrogen (TAN) often inhibits anaerobic digestion of mixed sludge. Lowering NH_3_ to non-inhibitory levels can improve digestion performance, enhancing chemical oxygen demand and volatile solids removal, which increases biogas production [28,29]. TAN levels between 1.5 and 7.0 g N L^−1^ can hinder anaerobic digestion [30]. This study investigated the effects of pH, temperature gradients, and receiving solutions on NH_3_ recovery and analyzed membrane integrity using atomic force microscopy (AFM) to examine surface morphology.

## 2. Materials and Methods

### 2.1. Synthetic Thermophilic Anaerobic Sludge

Synthetic sludge composition mimicked a real thermophilic anaerobic sludge and contained 5.0 g of sodium bicarbonate (NaHCO_3_), 0.85 g of potassium hydrogen phthalate (C_8_H_5_KO_4_), 1.70 g of ammonium chloride (NH_4_Cl), 0.90 g of urea (CO(NH_2_)_2_), 0.224 g of dipotassium phosphate (K_2_HPO_4_), 0.73 g of peptone from casein, 0.0175 g of sodium chloride (NaCl), 0.005 g of magnesium sulfate (MgSO_4_), and 0.01 g of calcium chloride (Ca_2_Cl) per liter of distilled H_2_O [31]. To achieve a specific pH, a solution of 12 M NaOH was used. All reagents were purchased from PANREAC (Panreac, Química S.A.U., Barcelona, Spain). The concentration of NH_3_ averaged 620.02 ± 4.18 ppm, while pHs averaged 7.82 ± 0.05.

### 2.2. Experimental Setup

**Figure 1:**
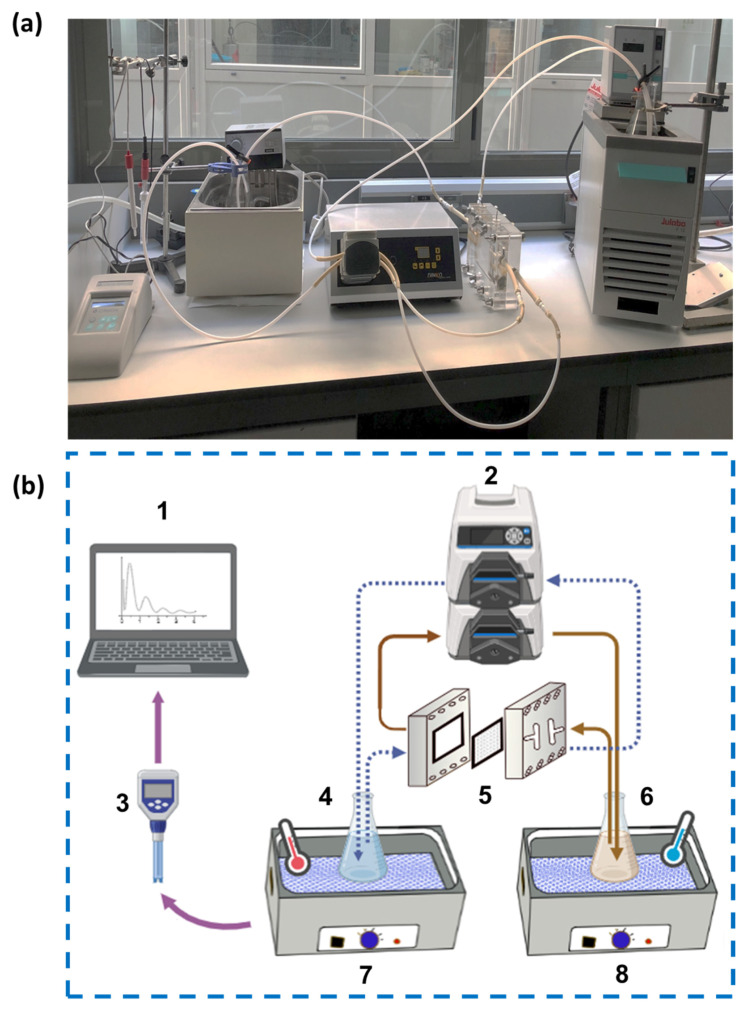
Photograph (**a**) and schematic representation (**b**) of the lab-scale ammonia recovery system by distillation. Computer (1), multiport peristaltic pump (2), pH meter (3), feed solution (4), membrane holder (5), receiving solution (6), thermostatic bath for heating (7), and thermostatic bath for heating and cooling (8).

The experimental setup used for the DCMD for NH_3_ recovery from thermophilic anaerobic sludges is shown in Figure 1. The synthetic thermophilic anaerobic sludge was continuously circulated at 0.25 L min ^−1^ using a multi-channel peristaltic pump (Dinko 25 VCF, DINKO instruments, Barcelona, Spain) over the active layer of a PTFE hydrophobic membrane with a nominal pore size of 0.22 µm (70% porosity, 150 contact angle, and 175 µm nominal thickness) manufactured by Millipore (Merck-Millipore, Burlington, MA, USA) in a customized cell module with a membrane area of 44 cm^2^ [32]. As receiving solution, deionized H_2_O and H_2_SO_4_ solution were recirculated at 0.25 L min ^−1^ on the support layer of the membrane using the same peristaltic pump. Both feed (synthetic thermophilic anaerobic sludge) and the receiving solutions (deionized H_2_O and H_2_SO_4_ solutions) were kept at different temperatures with two thermostatic baths (HAAKE type E12, Thermo Fisher Scientific, Waltham, MA, USA, and Julabo F12 Sigma-Aldrich, Merck, Steinheim, Germany) to achieve various temperature gradients in 0.5 L enclosed Erlenmeyer bottles.

### 2.3. Operational Conditions and Process Evaluation

Assays were conducted to elucidate the most suitable parameters for NH_3_ recovery by DCMD for synthetic thermophilic anaerobic sludge, using the described hydrophobic PTFE membrane. The assays were performed at different temperature gradients, receiving solutions, and pHs, with recirculation flow rates of 0.25 L min^−1^ (Table 1). In a first test series, preliminary assays were carried out with deionized H_2_O and 1 M H_2_SO_4_ as receiving solutions. In a second test series, assays were performed under temperature gradients of 0 °C (isothermal conditions) and 20 °C, using 1 M H_2_SO_4_ as the receiving solution. The third series simulated real thermophilic anaerobic sludge conditions using pH 8 and 1 M H_2_SO_4_ in the receiving solution. In a fourth test series, the feed solution was heated to 65 °C, with 1 M H_2_SO_4_ used as the receiving solution at pHs 8 and 12 under varying temperature gradients. The different experiments are summarized in Table 1.

After each experiment, the membrane system was washed twice for 1 h with deionized H_2_O. Feed solution samples were collected every 30 min over a 2 h period to analyze NH_3_ concentration and pH. Finally, a systematic evaluation of membrane integrity and deposition was performed by analyzing the membrane’s morphology at the end of the experiments. All experiments were conducted in triplicate. The standard deviation of these determinations, together with their scale error, allows us to quantify the total error of the magnitudes analyzed in this work.

### 2.4. Analytical Methods and Data Analysis

Temperature and pH were monitored using a Basic 20 pH meter with a 50 14T Crison electrode (Hach Lange Spain, Hospitalet de Llobregat, Spain). Total ammoniacal nitrogen (TAN) was measured using Nessler’s method at a wavelength of 425 nm in a Shimadzu UV-160A spectrophotometer at 425 nm (Shimadzu, Kyoto, Japan).

The theoretical flux of NH_3_ ($J_{NH_{3}}$) across the membrane was estimated using a vapor-phase transport model, assuming independent contributions from H_2_O and NH_3_ following Equation (1) [33]:

(1) $$J_{NH_{3}}=L_{NH_{3}}\left(p_{F,NH_{3}}-p_{C,NH_{3}}\right)$$

where $J_{NH_{3}}$ is the flux of NH_3_; $L_{NH_{3}}$ is the corresponding membrane permeability coefficient (mol m ^−2^ h^−1^ atm^−1^); and $p_{F,NH_{3}}$ and $p_{C,NH_{3}}$ the partial pressures of NH_3_ in the feed and the receiving solution, respectively. For assays using H_2_SO_4_ as the receiving solution, $p_{C,NH_{3}}$ was assumed to be zero due to complete chemical trapping of NH_3_. In contrast, when deionized H_2_O was used as the receiving phase, the concentration of NH_3_ in the collector was considered in the calculation of the driving force, either from direct measurement or estimated by mass balance.

The partial pressure of NH_3_ in the feed, $p_{F,NH_{3}}$, was calculated using Henry’s law (Equation (2)) [34], based on the mole fraction of dissolved NH_3_ and the vapor pressure of pure NH_3_ at the feed temperature. The mole fraction of NH_3_ was derived from the TAN concentration and pH, using the acid–base equilibrium between NH_4_^+^ and NH_3_:

(2) $$\left[NH_{3}\right]=\frac{TAN}{1+10^{\left(pK_{a}-pH\right)}}$$

where [TAN] is expressed in mol L^−1^, and $pK_{a}$ is the temperature-dependent dissociation constant of the NH_4_^+^/NH_3_ equilibrium. $pK_{a}$ values were obtained by NIST Chemistry WebBook [35]. The mole fraction of NH_3_ was then calculated by dividing the free NH_3_ concentration by the molar concentration of water mol L^−1^ and then multiplying by the vapor pressure of pure NH_3_ at 55 °C (0.796 atm) and 65 °C (1.238 atm) to obtain $p_{F,NH_{3}}$.

The flux of ammonium, $J_{NH_{3}},$ was obtained from the decrease in TAN concentration in the feed over 120 min, considering a feed volume of 0.5 L and a membrane area of 0.0044 m^2^:

(3) $$J_{NH_{3}}=\frac{\Delta n_{NH_{3}}}{A\Delta t}$$

where $\Delta n_{NH_{3}}$ is the number of moles of TAN lost from the feed between 0 and 2 h, and A is the membrane area, and Δt is 120 min.

The resulting values of $L_{NH_{3}}$ were then analyzed as a function of pH and the receiving solution. If consistent trends were observed, different values of $L_{NH_{3}}$ were used depending on the experimental conditions. Otherwise, an average value was used. Once permeability coefficients were determined, they were used to calculate the theoretical fluxes by substituting the corresponding $p_{F,NH_{3}}$ values into the flux equation.

To quantify the relative influence of each of the variables studied, a study was carried out by a three-way ANOVA with the software Statgraphics Centurion version 19.2.01.

### 2.5. Membrane Characterization Techniques

PTFE microporous membranes are commonly used in various membrane processes, and their structural characteristics have been accurately reported in the literature [36,37]. For this study, AFM analysis was added to test the membrane’s fouling in this specific application. AFM provides detailed information about the surface deposits and roughness on the membrane. AFM surface images were captured using a Nanoscope IIIA microscope in its Tapping mode (Digital Instruments, Veeco Metrology Group, Santa Bárbara, CA, USA) and analyzed using the NanoScope Software Version 5.30 (Veeco Metrology Inc., Santa Barbara, CA, USA).

## 3. Results and Discussion

### 3.1. Influence of the Capture and Dissolution of NH_3_

The pH and the type of receiving solution are key parameters of the DCMD process. Therefore, the effect of capture dissolution of NH_3_ under different pH conditions and temperature gradients is studied.

**Figure 2:**
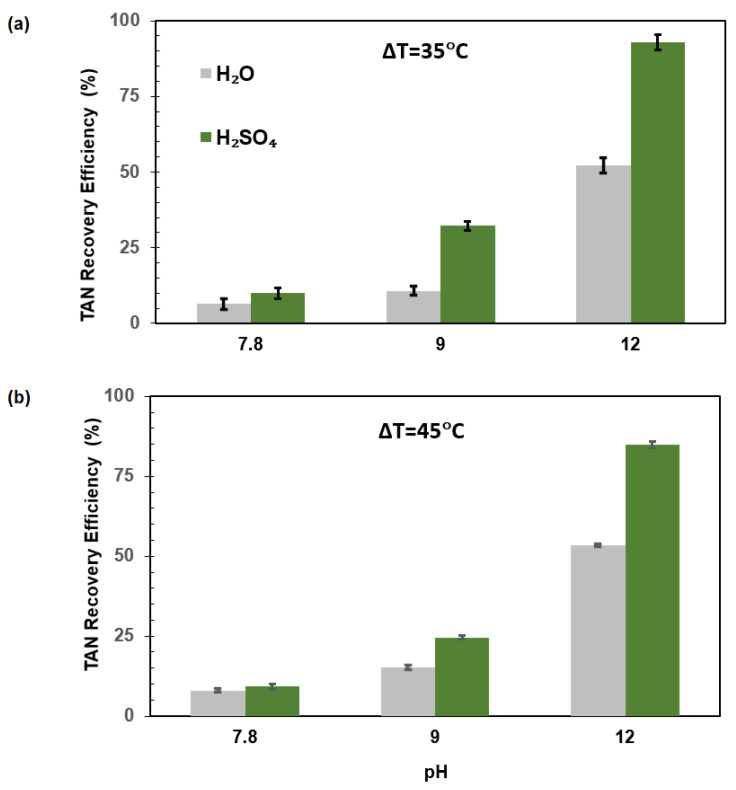
Influence of pH and the type of receiving solution on TAN recovery efficiencies at temperature gradients of 35 °C (**a**) and 45 °C (**b**).

The addition of NaOH to the feed solution decreases the proton concentration and increases the ionic strength of the system. These combined factors shift the NH_4_^+^/NH_3_ equilibrium towards NH_3_ formation. Consequently, an increased pH in the feed solutions resulted in enhanced NH_3_ recovery. In addition, the increase in ionic strength can inhibit methanogenic activity. However, in a previous study assessing the impact of pH on anaerobic digestion, the authors did not observe any significant effect on biogas production yields [38]. At higher pH values and a temperature gradient of 35 °C (Figure 2), NH_3_ recoveries improved significantly when using deionized H_2_O as a receiving solution. A similar trend was observed with 1 M H_2_SO_4_ as the receiving solution, although the recoveries were notably higher across the pH tested (7.8, 9, and 12). When the temperature gradient was increased to 45 °C, a further improvement in NH_3_ recovery was observed for H_2_O as the receiving solution, while a slight decrease in NH_3_ recovery was found with 1 M H_2_SO_4_ solution as the receiving solution (Figure 2).

In all assays, the combination of higher pHs and low temperature gradients contributed to enhancing the NH_3_ transfer from the digestate to the 1 M H_2_SO_4_ receiving solution. In all cases, this acidic receiving solution showed superior performance compared to deionized H_2_O. When a temperature gradient is established, the transport of species between the feed and the collector is strongly influenced by the nature of the fluid. In systems where H_2_O is present in the collector, its migration results in two detrimental effects: the dilution of NH_3_ in the receiving solution, which reduces the driving force for mass transfer, and the consumption of energy associated with the movement of H_2_O, thereby lowering the overall process efficiency. In contrast, when H_2_SO_4_ is used as the receiving solution, the behavior of the system changes significantly. It can be assumed that all the NH_3_ captured in the collector reacts to form ammonium sulfate ((NH_4_)_2_SO_4_), effectively reducing the vapor pressure of NH_3_ to zero. Although the entry of H_2_O into the collector still causes dilution and energy consumption, both considered undesirable, NH_3_ transfer is favored under these conditions. As expected, and as we will see later, the ammonia flow followed the same behavior as the NH_3_ recovery efficiency.

The NH_3_ concentration in the feed decreased with increasing collector temperature (it increased at lower temperature gradients, see Table 1), which caused NH_3_ concentration to remain low in the collector, sustaining a high concentration gradient that enhanced NH_3_ removal until full depletion in the feed was achieved. This outcome is beneficial, as it not only improves the efficiency of NH_3_ capture but also results in the direct formation of ammonium sulfate, adding value through simultaneous separation and fertilizer production. Previous work reported TAN recoveries of 84% after 3.5 h of operation using a membrane contactor with a receiving solution of 1 M H_2_SO_4_ at 35 °C [37]. Moreover, a similar study reported a 98% recovery of nitrogen in the form of free NH_3_ using vacuum MD from biogas slurry with hollow fiber PVDF membranes at 35 to 37 °C [39].

pH and temperature are crucial environmental factors during NH_3_ extraction in membrane-based processes since both parameters govern the mass transfer of NH_3_ throughout the membrane. The increase in pH in the feed solution caused a positive effect on NH_3_ removal during the DCMD of thermophilic anaerobic sludge carried out in this work. Previous studies demonstrated that the operation time, feed concentration, and pH are significant factors influencing NH_3_ recovery [40]. NH_3_ recovery was strongly influenced by both the temperature and the presence of a temperature gradient, with distinct behaviors observed depending on the nature of the receiving solution. Thus, when H_2_O was used as the absorbent, recoveries increased with temperature and gradient, in accordance with the expected rise in NH_3_ volatility and vapor pressure at elevated feed temperatures. The increased thermal gradient likely enhanced mass transfer by promoting NH_3_ diffusion across the membrane. However, due to the limited NH_3_ trapping capacity of H_2_O and the potential back diffusion or re-volatilization of NH_3_, the overall recovery remained moderate under these conditions. In contrast, when H_2_SO_4_ was employed as the receiving solution and the feed pH was adjusted to 12, recoveries were substantially higher across all conditions. In this configuration, NH_3_ readily reacted with the acid to form non-volatile ammonium sulfate, maintaining an effectively zero NH_3_ partial pressure on the permeate side. This created a strong and sustained driving force for NH_3_ transfer, independent of the temperature gradient. Interestingly, although higher gradients slightly reduced recovery efficiency, this effect may be attributed to the dilution of acid or energy losses associated with increased water flux, rather than limitations in NH_3_ transport. Overall, the results highlight the dominance of chemical capture mechanisms in controlling recovery efficiency, with temperature acting as a facilitator of volatilization rather than the primary driving factor when strong acid trapping is present. In the absence of a temperature gradient and with H_2_SO_4_ as the absorbent in the collector, the system exhibits distinct behavior characterized by several favorable effects. Under these conditions, it can be assumed that all the NH_3_ present in the collector reacts to form ammonium sulfate ((NH_4_)_2_SO_4_), resulting in an NH_3_ vapor pressure effectively equal to zero. The elimination of the temperature gradient also removes the vapor pressure gradient of H_2_O, thereby preventing H_2_O transfer into the collector. As a result, dilution of the captured NH_3_ is avoided, and no additional energy is consumed due to fluid movement, both considered positive outcomes. Regarding NH_3_ transfer, the concentration in the feed decreases with temperature, while the concentration in the collector remains zero. This establishes a strong and sustained driving force for NH_3_ transport, which persists until complete depletion of NH_3_ in the feed solution is achieved. Moreover, the direct formation of ammonium sulfate within the collector represents an added advantage by enabling simultaneous ammonia removal and fertilizer production under energy-efficient conditions. In Section 3.3, we will see that these trends are predicted with a simple mass transfer model like the one outlined in Section 2.4. Previous studies reported 20% TAN recovery through DCMD of synthetic urine with a pH of 8.8 and a temperature gradient of 40 °C [41]. Conversely, the current work used 1 M H_2_SO_4_ as the receiving solution and obtained TAN recoveries ranging from 13.7% to 39.0% at pH values of 7.8 and 9.0, with temperature gradients of 0 and 20 °C.

### 3.2. Effect of System Conditions Using H_2_SO_4_ as a Receiving Solution Recovery

**Figure 3:**
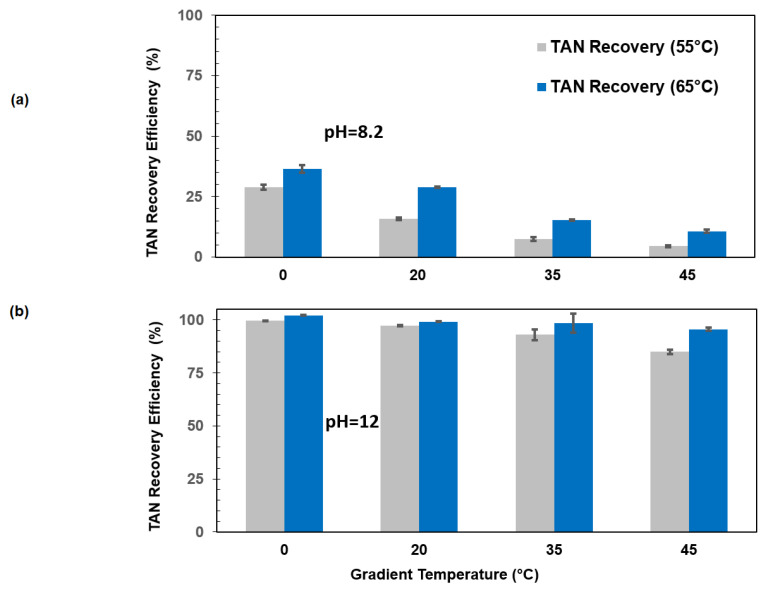
TAN recovery efficiencies and for pHs 8.2 (**a**) and 12 (**b**) with temperature gradients of 0 °C, 20 °C, 35 °C, and 45 °C for feeds with 55 °C and 65 °C, having H_2_SO_4_ 1 M at the permeate side.

Considering the higher efficiency of acid-based capture, the influence of the operational parameters, such as temperature gradient and feed temperature, on the NH_3_ capture process should be studied in greater detail when using H_2_SO_4_ as the receiving solution. This detailed analysis is crucial as far as TAN extraction must be carefully controlled, because methanogenesis strongly depends on the NH_3_ concentrations [42,43]. In this case, the feed solution was adjusted to pH 8.2 to simulate thermophilic anaerobic sludge at both 55 °C and 65 °C, while the receiving solution was 1 M H_2_SO_4_, with temperature gradients of 0 °C, 20 °C, 35 °C, and 45 °C (Figure 3).

The effect of temperature on NH_3_ recovery was found to be significant across all experimental conditions (Figure 3). Higher feed temperatures enhanced NH_3_ volatilization, increasing its partial pressure and promoting mass transfer across the membrane. This behavior is consistent with the thermodynamic prediction of an increase in ammonia’s volatility with temperature, thereby improving its separation efficiency. Additionally, the presence or absence of a temperature gradient played a key role in the system’s performance. In configurations without a thermal gradient, where the feed and the receiving solution were maintained at the same temperature, NH_3_ recovery was notably improved when H_2_SO_4_ was used as the absorbent. This can be attributed to the elimination of water flux into the collector, which otherwise causes dilution and energy loss. Furthermore, maintaining a high pH in the feed solution increased the proportion of un-ionized NH_3_, which is the volatile species responsible for nitrogen mass transfer, thus facilitating NH_3_ capture. Overall, the best performance was observed under conditions of high feed temperature, no temperature gradient, and elevated pH, an optimal combination that maximized NH_3_ recovery and minimized energy loss.

While high feed temperatures, absence of a thermal gradient, and elevated pH were found to optimize NH_3_ recovery, it is important to consider the potential drawbacks of operating under such conditions, particularly in systems where anaerobic digestion is integrated. Methanogenic microorganisms, responsible for methane production, are highly sensitive to temperature. Most thrive within the mesophilic range (35–40 °C), and their metabolic activity can be significantly inhibited at higher temperatures unless the system is specifically adapted to thermophilic conditions (typically 50–60 °C). Operating the feed stream at elevated temperatures may therefore disrupt microbial homeostasis, reduce biogas yield, and compromise process stability. Moreover, prolonged exposure to thermal stress may lead to shifts in microbial community structure or even cell lysis, negatively affecting methane production efficiency. To address this tradeoff, several strategies can be employed. One approach involves decoupling the ammonia stripping and anaerobic digestion stages, allowing the digestate to be treated thermally in a separate unit without exposing the microbial community to high temperatures. Alternatively, side-stream treatment of a fraction of the digestate can be implemented to selectively remove ammonia while maintaining favorable conditions in the main reactor. Heat recovery systems can also be integrated to improve energy efficiency and reduce operational costs. Additionally, if thermophilic digestion is considered, microbial adaptation and careful process control must be ensured to maintain a stable and active microbial consortium. These strategies provide pathways to optimize ammonia removal without compromising the biological performance of methane-producing systems. However, in this specific case at pH 12, the decrease in recovery efficiency when lowering the temperature from 65 °C (100%) to 55 °C (99.6%) is minimal. The system appears to be much more sensitive to the pH value. In this case, it would be necessary to assess whether it is feasible to design a process that includes stages of basification, distillation, and re-acidification.

Numerous benefits make isothermal distillation a highly effective method for a range of commercial and laboratory uses. One of its primary advantages is that it operates more simply since it does not require complicated temperature control systems, which lowers costs and the complexity of operation [44]. Additionally, it uses less energy since it does not involve the constant heating or cooling that other distillation processes require [45]. By enabling more accurate separation of volatile chemicals, isothermal distillation increases separation efficiency and improves product purity [46]. Furthermore, a constant temperature helps in the regulation of reaction kinetics, which is crucial when working with delicate materials that may deteriorate in the presence of temperature fluctuations [47]. Finally, isothermal distillation provides consistent product production and quality, making it a dependable and energy-efficient option for a variety of applications [48].

### 3.3. Statistical Analysis

The ANOVA analysis revealed that the most significant effect was attributable to the receiving solution on NH_3_ recovery (F(1,8) = 295.84, *p* < 0.0001). Additionally, there was a significant interaction between feed pH and receiving solution (F(3,8) = 26.23, *p* = 0.0002), and between receiving solution and temperature gradient (F(3,8) = 8.73, *p* = 0.0066). Neither feed pH alone nor the three-way interaction was significant (*p* > 0.05), indicating that the effect of pH depends on the type of receiving solution but not significantly on the temperature gradient when all three factors are considered together.

### 3.4. Validation of Theoretical Flux Predictions with Experimental Data

NH_3_ fluxes across the membrane were evaluated using both experimental observations and theoretical predictions. Experimental values were obtained from the measured decrease in TAN in the feed solution over time, normalized by the membrane area and duration of each assay, following established procedures in NH_3_ recovery studies. Theoretical fluxes (J_T_) were estimated using a vapor-phase transport model based on the partial pressure difference of ammonia between the feed and receiving compartments, briefly described in Section 2.4. The method has been widely applied in membrane distillation and gas-permeable membrane systems. The partitioning of TAN into NH_3_ and ammonium was calculated from pH and temperature-dependent dissociation constants, as described in previous studies. This comparison between predicted and experimental fluxes (J_E_) was used to assess model performance and to explore the influence of operational parameters such as pH, temperature gradient, and the nature of the receiving solution on NH_3_ transport efficiency.

**Figure 4:**
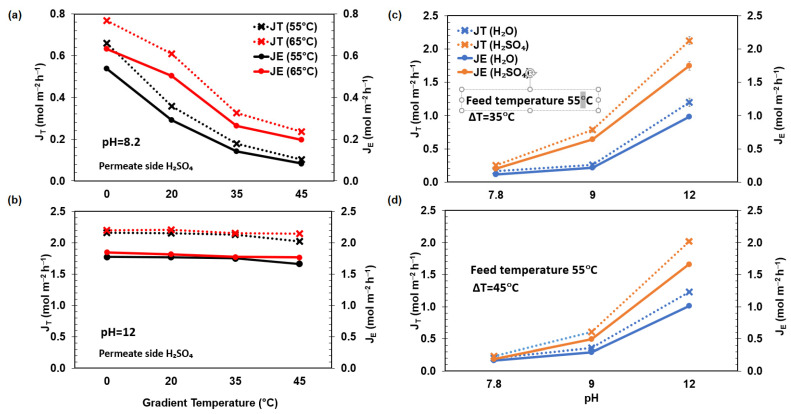
Comparison of theoretical and experimental NH_3_ molar fluxes versus temperature gradient, (**a**,**b**), having H_2_SO_4_ 1 M at the permeate side; and versus pH, (**c**,**d**), having H_2_O and H_2_SO_4_ at the permeate side.

The comparison of J_T_ and experimental J_E_ molar NH_3_ fluxes (Figure 4) demonstrates a strong dependence on both feed solution pH and the applied temperature gradient. An increase in pH from 7.8 to 12 resulted in a substantial rise in NH_3_ fluxes, attributable to the shift in ammonia speciation toward the volatile NH_3_ form, which enhances the driving force across the membrane. This trend is accentuated when 1 M H_2_SO_4_ is used in the permeate phase, promoting efficient acid trapping and sustaining a low NH_3_ partial pressure on the permeate side. In contrast, increasing the temperature gradient from 0 °C to 45 °C led to a systematic decline in both J_T_ and J_E_, particularly under isothermal feed conditions at 55 °C and 65 °C. This reduction is likely associated with enhanced transmembrane water vapor flux, which induces concentration polarization at the feed interface and may reduce local NH_3_ partial pressures, limiting the effective driving force. Although elevated feed temperatures improved ammonia fluxes at constant gradients due to increased NH_3_ volatility, this effect diminished at higher gradients, indicating competing transport phenomena. Across all experimental conditions, theoretical fluxes overpredicted the measured values, highlighting the influence of non-idealities such as interfacial mass transfer limitations, pore diffusion resistance, and liquid phase polarization effects not accounted for in the simplified transport model. Previous work reported a molar flux of 4.52 mol TAN m^−2^ h^−1^, after 3.5 h of operation using a membrane contactor with a receiving solution of 1 M H_2_SO_4_ at 35 °C [37]. A similar approach reported a molar flux of 0.18 mol TAN m^−2^ h^−1^, using a stripping process using hollow fiber PVDF membrane configuration at 25 °C for simulated wastewater at pH 10 [49]. Hu and coworkers (2024 b) reported an NH_3_ recovery efficiency of 98.72% which matched their own model prediction and was statistically proven with ANOVA [40].

Equation (1) does not account for the volatilization of NH_3_ in the feed or its condensation in the receiving phase, both of which may limit the actual transport rate and lead to overestimation of fluxes. This discrepancy can arise from several phenomena. First, the membrane interfaces may not be at thermodynamic equilibrium due to resistance within the pores, causing the effective partial pressure of NH_3_ at the feed side to be lower than predicted, and at the collector side to be higher. Additionally, under isothermal conditions, back diffusion of water vapor can interfere with NH_3_ transport through molecular collisions, effectively reducing the net flux, as already mentioned. Finally, concentration polarization effects in the liquid phases may lead to differences between bulk and interfacial concentrations, diminishing the local driving force for mass transfer.

### 3.5. Membrane Morphology Analysis

**Figure 5:**
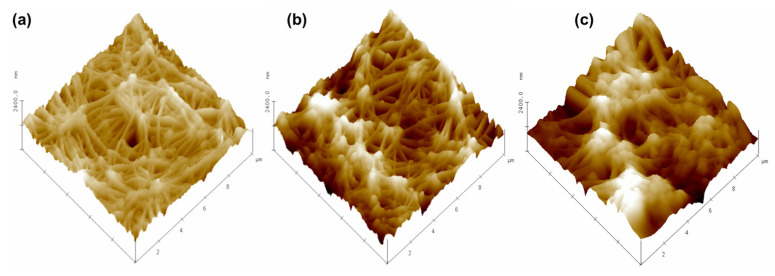
AFM 3D topographic images of the new membrane (**a**), active layer of the used membrane (**b**), and support layer of the used membrane (**c**) (scanned area 10 µm × 10 µm).

Figure 5 depicts three-dimensional AFM images of the used membranes when pristine and after their use under multiple operational conditions and assays. In the active layer of the membrane, which is in contact with the feed solution, deposition was detected. On the support layer in contact with the 1 M H_2_SO_4_ solution, which functions as the receiving solution, the acid deteriorates and modifies the original fibrous structure of the membrane.

To quantify the effects observed in the images, the roughness, $R_{q}$ (Equation (4)), was determined.

(4) $$R_{q}=\sqrt{\frac{\sum {\left(Z_{i}\right)}^{2}}{N}}$$

where $Z_{i}$ is the current *Z* coordinate height value, and N is the number of points.

Table 2 shows the average values obtained for the roughness of both sides of the used membrane and the clean membrane. Here, the bearing volume includes the total volume over the mean height plane.

It is observed that, because of the fouling (deposition of substances on the surface), the roughness of the active layer increased. Considering the composition of the feed solution (see Section 2.1), it is most likely that most of the deposited substances are short-chain peptides from peptone, along with some of the organic salts present, due to their hydrophobic character and affinity for the membrane material. By contrast, the acid attack on the support side decreased the roughness: the acid attacks protuberances, smoothing the surface. Similar studies performed by Zhang et al. (2015) reported that the unused membrane has a topography with greater roughness compared to the used membrane [50].

To corroborate this finding, the bearing volume was determined, i.e., the volume of the sample above the mean height (calculated as 50% of the bearing analysis surface). The results obtained are shown in Table 2. These results show that the active layer of the fouled membrane accumulates a substance from the solution, while the support layer loses membrane material due to the acid intrusion. However, it is worth mentioning that both these modifications are low. Considering that PTFE is inert to sulfuric acid under these working conditions, and given that the membrane achieves its porous structure through the biaxial stretching process, it is plausible that this manufacturing method generates structural defects or microfractures within the polymer. These imperfections could be the cause of the material loss observed after prolonged exposure to sulfuric acid. However, previous studies show the overall integrity of the membrane for times greater than 160 days [25].

Previous research has indicated that fouling layers contribute to a decrease in membrane hydrophobicity, ultimately resulting in reduced efficiency of NH_3_ extraction [51,52]. To address this issue, it would be essential to employ chemical and physical cleaning methods to alleviate the effects of fouling on membrane performance [53,54].

## 4. Conclusions

MD mediated a significant improvement in the process of NH_3_ recovery from digestates. This approach was enhanced when increasing pH, operating at isothermal conditions, and having as a receiving solution 1 M H_2_SO_4_. NH_3_ recoveries of 100.00 ± 0.11% were recorded in the synthetic thermophilic anaerobic sludge under these operational parameters. In addition, it allows obtaining (NH_3_)_2_SO_4_ as a fertilizer. Process operation at pH values of 12 supported the highest NH_3_ recoveries. Membrane fouling and deterioration were not relevant according to AFM results. Overall, MD represents a promising technique for NH_3_ recovery that combines efficiency, environmental sustainability, and economic feasibility. MD implementation in wastewater treatment and resource recovery can make a major contribution to reducing nitrogen pollution and recovering valuable resources. However, further studies will be necessary for the engineering design of the process, taking into account factors such as fouling and membrane integrity, operating times, water vapor flux, etc., when treating actual thermophilic digestate.

## Figures and Tables

**Table 1:** Operational conditions used in the optimization of DCMD.

Series	Assay	Feed pH	Feed Temp. (°C)	Receiving Solution	Receiving Solution Temp. (°C)	Temp. Gradient (°C)
I	1	7.8	55	DI H_2_O	20	35
	2	7.8	55	H_2_SO_4_	20	35
	3	7.8	55	DI H_2_O	10	45
	4	7.8	55	H_2_SO_4_	10	45
	5	9	55	DI H_2_O	20	35
	6	9	55	H_2_SO_4_	20	35
	7	9	55	DI H_2_O	10	45
	8	9	55	H_2_SO_4_	10	45
	9	12	55	DI H_2_O	20	35
	10	12	55	H_2_SO_4_	20	35
	11	12	55	DI H_2_O	10	45
	12	12	55	H_2_SO_4_	10	45
II	1	7.8	55	H_2_SO_4_	55	0
	2	7.8	55		35	20
	3	9	55		55	0
	4	9	55		35	20
	5	12	55		55	0
	6	12	55		35	20
III	1	8.2	55	H_2_SO_4_	55	0
	2	8.2	55		35	20
	3	8.2	55		20	35
	4	8.2	55		10	45
IV	1	8.2	65	H_2_SO_4_	65	0
	2	8.2	65		45	20
	3	8.2	65		30	35
	4	8.2	65		20	45
	5	12	65		65	0
	6	12	65		45	20
	7	12	65		30	35
	8	12	65		20	45

**Table 2:** Roughness and bearing volume obtained from AFM images.

Membrane	$R_{q}\;$ (nm)	Bearing Volume (μ^3^)
New membrane	200 ± 8	9400 ± 90
Used membrane (Active layer)	246 ± 25	11,800 ± 130
Used membrane (Support layer)	147 ± 6	5000 ± 50

## Data Availability

The original contributions presented in this study are included in the article. Further inquiries can be directed to the corresponding authors.
